# An empirical study on the development of metaphorical comprehension of Chinese children

**DOI:** 10.3389/fpsyg.2023.1254129

**Published:** 2024-01-08

**Authors:** Lulu Cheng, Yingming Guan, Ting Zhang, Linlin Zhan, Yanqin Liu, Peng Wang, Shanshan Yu, Yule Peng

**Affiliations:** ^1^Shanghai Center for Research in English Language Education, Shanghai International Studies University, Shanghai, China; ^2^School of Foreign Studies, China University of Petroleum (East China), Qingdao, China; ^3^Journal of Tianjin Normal University (Social Science Edition), Tianjin, China; ^4^College of Engineering, Faculty of Information and Engineering Science, Peking University, Beijing, China; ^5^School of Western Studies, Heilongjiang University, Harbin, China; ^6^Department of Language, Literature and Communication, Faculty of Humanities, Vrije Universiteit Amsterdam, Amsterdam, Netherlands; ^7^Department of Psychology, Education and Child Studies, Erasmus School of Social and Behavioural Sciences, Erasmus University Rotterdam, Rotterdam, Netherlands; ^8^College of Foreign Languages, Ocean University of China, Qingdao, China

**Keywords:** Chinese children, metaphorical comprehension, salient, behavioral experiments, language development

## Abstract

Metaphor affects how people focus, remember, and process information and significantly influences children’s language development. The study explored metaphorical comprehension by Chinese children of different ages (5–8 years). We collected response times and accuracy rates when they processed metaphorical and literal sentences with the graded salience. Linear mixed-effects modeling showed that Chinese children’s metaphorical ability improved with age. Subsequent analysis found that the perception period of metaphorical knowledge was at age 5, the development stage of metaphorical knowledge was at age 6 and 7, and the rational decision period of metaphorical ability was at age 8. After 8-year-old, children can invoke the knowledge of the intention schema while activating the source domain, and this knowledge can be automatically and quickly mapped to the target domain. Meanwhile, language development and cognitive processing influenced the metaphorical comprehension of Chinese children, especially children of 8 years of age who had the highest correct rate and the shortest reaction time to process low-saliency metaphorical sentences, while 5-year-old children had the highest accuracy in high-saliency metaphorical sentence and 6-year-old children got the longest reaction time to process sentence in high-saliency metaphor. This study may provide evidence for improving and training metaphor comprehension in children with special needs such as those with an autism spectrum disorder.

## 1 Introduction

Metaphors pervade daily discourse, serving as a pivotal mechanism in both communication and cognitive processes. By furnishing a tangible structure for abstract concepts, metaphors exert a profound influence on the modalities of attention, retention, and information processing (Boers, 2000; Fernandez-Duque and Johnson, 2002; Iskandar and Baird, 2014; Thibodeau et al., 2017; Ahrens and Gong, 2021; Li et al., 2022). This linguistic phenomenon underscores the intrinsic role of metaphorical constructs in shaping conceptual frameworks and facilitating cognitive operations. For instance, the characterization of a lawyer as a ‘shark’ exemplifies a metaphoric transference that is not readily apparent, bridging disparate ontologies—that of the legal professional and the predatory fish. This metaphorical conflation supports the argument that metaphors are entrenched not solely within the domain of language but extend their influence into the realms of thought and action (Lakoff and Johnson, 1980; Gibbs, 1994; Gibbs and Tendahl, 2006; Kövecses, 2010; Gibbs, 2013; Alessandroni, 2017), serving as a strong evidence for a metaphorical conceptual system highly grounding on a linguistic basis.

The seemingly effortless generation and comprehension of metaphors have garnered escalating scholarly attention, as evidenced by the works of Glucksberg and Keysar (1993), Landau et al. (2015), Al-Azary and Katz (2021), and Carston and Yan (2023). This growing body of research has also gradually substantiated metaphor as a concept of cognitive mechanism (Bai, 2004; Pouscoulous and Tomasello, 2020). Empirical studies, such as those by Sperber and Wilson (1991), further validate the intrinsic and ubiquitous manifestation of metaphorical thought in children’s language, suggesting an innate metaphorical competence evidenced through their spontaneous metaphorical expressions. Children’s grasp of metaphorical concepts exemplifies the cognitive-linguistic interface where experiential phenomena are mapped from a source to a target domain. Here the target domain refers to the starting point or the described concept of the metaphor, which is the cognitive concrete category or abstract category; While the source domain is the specific category, which is used to compare the target domain. It is due to the existence of cross-domain mappings that we can think and talk about one domain, which is Conceptual Metaphor Theory (Coats, 2019; Conrad and Libarkin, 2022; Maretha and Wahyuningsih, 2023). This mapping evidences the idiosyncratic manner in which children actively engage in the creative depiction of reality, thereby contributing to their epistemic construction. Such metaphorical mappings are indicative of the child’s developing capacity to abstractly relate different domains of knowledge and to articulate their understanding of the world (Wiśniewska-Kin, 2023). As a crucial aspect of communicative competence, children’s metaphorical competence refers to their ability to understand, interpret, and use metaphors effectively in communication by successfully perceiving of space, identifying and analyzing the conceptual mapping. It involves the skill of recognizing and comprehending the underlying meaning and symbolism conveyed through metaphors (Özcaliskan, 2005; Sabet, 2016).

Thus, metaphorical competence is an important embodiment of children’s experience of the world and internalization of knowledge. Current evidence has shown that the metaphorical competence, cognitive flexibility, and information processing speed increase along with children’s development (Willinger et al., 2017; Yuan, 2020). It has been reported that the early cognitive and linguistic ability in metaphor comprehension was first charted in children’s age between 3 and 5 (Wellman, 1990; Özcaliskan, 2005). They have already demonstrated the ability to interpret the keywords in metaphorical context at 7 years old, and this ability is further strengthened when they are 9 (Nippold et al., 1984), evidencing the positive association between metaphorical competence and age. In this sense, children’s metaphorical understanding ability constantly grows over time, and the capability of proactive utilization of metaphor will also be acquired at later stages.

Prevailing research posits that even children as young as 3 years old exhibit the capacity for metaphorical comprehension, and this metaphorical competence appears to augment as they age. Despite these advancements in understanding the developmental trajectory of metaphorical cognition, the intricate cognitive processes underpinning the development of children’s metaphorical abilities remain to be fully elucidated by empirical research. Researchers have focused on examining children’s understanding of the temporal domain and some conceptual domains (Zhou and Huang, 2001; Liu and Mi, 2008; Du et al., 2020) or on case studies of children’s metaphorical output abilities and illustrating them with cross-sectional data (Pan and Zhou, 2018, 2021), as well as on the characteristics and developmental patterns of children’s metaphorical thinking (Bai, 2004). Previous literature has demonstrated inconsistent findings regarding children’s early metaphorical capability. On the one hand, it has been indicated that the evidence suggests that the literal meaning is better understood than metaphorical ones because of children’s generally weaker ability to understand abstract relations (Winner et al., 1980; Silberstein et al., 1982; Gentner, 1988; Zhou and Huang, 2001; Bai, 2004; Liu and Mi, 2008; Pan and Zhou, 2018, 2021; Du et al., 2020). What should be paid specific attention to is the effect the conventionality of metaphor may have in the processing speed of metaphor and literal meaning. Considering the nature of conventional metaphor, which is “the ordinary conceptual system reflected in our everyday language (Lakoff and Johnson, 1980),” the current study would ignore the effect of conventional metaphor as the repeated exposure has made it approximately equal to literal sentences (Prabhakar et al., 2018). While on the other hand, previous research has also reported balanced development of some children’s metaphorical and literal language production and comprehension ability, supported by the excellence in reasoning abstract relations (Gentner, 1977; Inhoff et al., 1984; Keil, 1986; McElree and Nordlie, 1996; Walker and Cooperrider, 2016). The temporal dynamics of metaphorical meaning activation remain contentious, particularly regarding whether metaphor comprehension is mediated by direct or indirect cognitive processes (Glucksberg, 2001, 2003, 2008; Wackers et al., 2021; Steen, 2023). The traditional indirect model posits a two-stage processing approach where metaphors are initially interpreted literally, and upon encountering difficulty or incongruity, a specialized metaphorical processing system is engaged to infer pragmatic meaning (Searle, 1979; Genovesi, 2020; Pissani and de Almeida, 2023). This model suggests that metaphor comprehension is a more laborious, secondary process compared to literal interpretation.

Contrastingly, contemporary studies have posited that children’s metaphorical cognition, informed by Theory of Mind, may operate without the additional effort delineated in the classical model. Theory of Mind, which encompasses the prediction and manipulation of mental states based on cultural and social knowledge, appears to facilitate metaphorical understanding from an early age (Wellman, 1990; Frith and Frith, 2003; Norbury, 2005; Lecce et al., 2014). This cognitive ability enables children to navigate the abstract and symbolic nature of metaphorical language, aligning with speakers’ intentions and shared cultural contexts, thereby suggesting that metaphorical cognition could be as immediate as literal meaning comprehension.

However, there is a noticeable paucity of studies exploring metaphorical competence in Chinese children, with limited exploration into how they process temporal, conceptual domains, and generate metaphorical expressions (Zhou and Huang, 2001; Liu and Mi, 2008; Pan and Zhou, 2018, 2021; Du et al., 2020). This represents a significant gap in our understanding of cross-cultural cognitive development in metaphor comprehension.

Research methodologies on children’s metaphorical competence vary widely, encompassing techniques such as task-based language assessments, pictorial description tasks, lexical gap-filling, and narrative comprehension exercises. These methods also include verbal reporting, naturalistic observation, and structured interviews (Nippold et al., 1984; Özcaliskan, 2005; Rundblad and Dimitriou, 2010; Li, 2011, 2012; Kuang and Zhou, 2018; He et al., 2021; Pan and Zhou, 2021; Sun, 2021). However, the integration of these diverse methods into a cohesive framework that provides behavioral metrics for metaphor comprehension has been limited.

Depending on cognitive development, metaphor provides a way of categorizing reality (Carriedo et al., 2016; Pastor et al., 2020). Utilizing quantitative and descriptive behavioral experiment, the current study aimed to explore the underlying cognitive process of metaphorical comprehension. To be specific, the reaction time (RT) and accuracy rate (ACC) measured by behavioral experiment during the meaning decision task employed among children at different ages have been used to reflect the cognitive process indirectly. This is poised to deliver a more comprehensive understanding of the developmental patterns in children’s metaphor comprehension abilities, including potential individual differences across varying age groups.

In conclusion, each work focuses on an aspect to be investigated and on a single paradigm. Nevertheless, they all aim at better understanding when and how metaphor comprehension skills appear in children. However, there exists relatively little research on the evolution of metaphorical comprehension among Chinese children. This study is poised to delineate how Chinese children across different age brackets differentially process metaphorical and literal sentences, employing a sophisticated behavioral experiment framework. Therefore, the goal of this study is to solve this problem by investigating the temporal and accuracy-related aspects of metaphor processing but also elucidating the underlying cognitive neural mechanisms. By doing so, it seeks to contribute to a more granular understanding of the temporal dynamics in metaphor acquisition and to enhance the empirical basis for cognitive theories of language processing.

## 2 Methodology and materials

First, 40 literal and 40 metaphorical sentences were selected from children’s early years picture books, classic story theater series, 100 nursery rhymes, and other related books^1^ familiar to children. Both metaphorical and literal sentences have same structure: ‘A is B.’ In the metaphorical cases, A is understood or explained by B (e.g., “The rainbow is an arch bridge. (The shape of rainbow is like an arch bridge)” “彩虹是拱桥”), involving a mapping of two conceptual domains (Shu, 2004, p. 27). For ‘A is B,’ A is the starting word, which is the core of the meaning of the whole sentence, and is usually the nominative absolutive noun; while B as a predicate, is the component to explain “what is A” or “How is a.” From this perspective, copular constructions in Mandarin are identical to English copular constructions.

Second, in this study, the salience of metaphorical and literal sentences was systematically evaluated, with salience defined as the immediacy of a sentence’s meaning as it is perceived by a reader or speaker (Giora, 1997). This construct was operationalized through four indicators: conventionality, familiarity, prototypicality, and frequency, which together facilitate the classification of sentences into categories indicative of high or low cognitive prominence (Giora, 2003; Lai et al., 2009). That is to say, meanings of words, phrases, or sentences (e.g., the conventional interpretations of idioms or provers) have to be coded in the mental lexicon and, in addition, enjoy prominence due to a meaning is more widely and frequently used in a linguistic community, a more prototyping meaning, a more familiar or recently acquired meaning. Meanings not coded in the mental lexicon (e.g., conversational implicatures constructed on the fly) are non-salient. In addition, according to the method recommended by Grioa, metaphorical and literal sentences with high or low-saliency levels were selected in this study.

To empirically measure these dimensions of salience, we adopted a robust experimental design informed by Zhou (2011), which included the enlistment of 6 seasoned educators to appraise our sentences. Utilizing a five-point Likert scale, these educators assessed each sentence for familiarity, conventionality, and prototypicality. The mean scores from these assessments were used to assign a salience level to each sentence, leading to a bipartite categorization: sentences of high salience and those of low salience within both metaphorical and literal classifications. The assessments by these domain experts provided the basis for the nuanced categorization of the study’s sentences, facilitating a rigorous examination of salience as it relates to metaphor comprehension.

In addition, we randomly selected 20 non-experimental control participants (5–8 years old age group, 5 in each group to rate the familiarity of 80 sentences.^2^ In order to ensure that children understand the meaning of metaphor, we employed a meaning decision task, in which each type of sentence including filler sentences has been tested. Furthermore, children are asked to verbally explain the key words that indicate metaphor in the sentence. In this study, the sentences with mean scores less than 2.5 was removed. Because the total score is 5, less than half of which needs to be deleted. Finally, 48 experimental sentences remained, including 24 metaphorical sentences and 24 literal sentences. The composite scores of metaphorical and literal sentences in the top half are high-saliency sentences, while composite scores belong to the bottom half are low-saliency sentences. In addition, another 24 filler sentences were used to complete meaning decision task, 12 high-saliency metaphorical sentences, 12 low-saliency metaphorical sentences, 12 high-saliency literal sentences, and 12 low-saliency literal sentences are also included. The results of the variance analysis showed that there was a significant difference between high-saliency metaphorical sentences and low-saliency metaphorical sentences in terms of salience (*p* < 0.001), between high-saliency literal sentences and low-saliency literal sentences in terms of salience (*p* < 0.001). There was no significant difference between high-saliency metaphorical sentences and high-saliency literal sentences in salience (*p* = 0.903) and no significant difference between low-saliency metaphorical sentences and low-saliency literal sentences in terms of salience (see Table 1).

**Table 1:** The mean of salience according to the sentence type (standard deviation in parentheses).

**Condition**	**Example**	**Salience**
High-saliency metaphorical sentences	星星是眼睛 (Stars are eyes.)	4.647 (0.220)
Low-saliency metaphorical sentences	彩虹是拱桥 (The rainbow is an arched bridge.)	3.941 (0.210)
High-saliency literal sentences	婴儿是孩子 (Babies are children.)	4.653 (0.109)
Low-saliency literal sentences	认真是前提 (Seriousness is the prerequisite.)	4.200 (0.198)

Finally, in order to complete the meaning decision task, 24 filler sentences were also added to this study. The filler sentence has the same pattern as the experimental sentence, but the semantics are violated. For example, ‘the building is fish’. The study includes the following research questions: (1) The periods children (aged 5–8)’s metaphorical competence can be divided into, and the specific development characteristics of children (aged 5–8)’s metaphorical competence; (2) The influencing factors of metaphorical comprehension of Chinese children. Based on the results of previous studies, the study speculated that the metaphorical competence of children (5–8 years old) shows an increasing trend with the growth of age, and the development of metaphorical competence of children at different ages has certain differences.

### 2.1 Participants

Forty children aged 5–8 years^3^ whose native language was Chinese were selected to participate in this experiment. There were 12 children in each age group, with half of them being boys and half being girls.^4^ All participants were in good health, had normal or corrected vision, were right-handed and had no history of psychiatric or neurological disease, traumatic brain injury, or other related medical conditions. Participants and their parents or legal guardians signed a protocol. Furthermore, the study protocol was approved by the medical ethics committee of the university of researchers.

### 2.2 Experimental procedure

**Figure 1:**
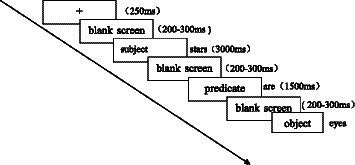
Schematic diagram of the experimental process.

In this study, an experimental program for sentence comprehension was written using E-prime 2.0. The sequence of the experimental procedure consisted of presenting the gaze point “+” (SONG, 40)^5^ in the center of the screen for 250 ms, followed by a random blank screen for 200–300 ms. Then, the subject of a sentence (SONG, 40, two-character word) was presented for 3,000 ms. And the predicate of a sentence (SONG, 40, single character) was presented for 1,500 ms after a random blank screen from 200 to 300 ms. After that, a random blank screen was still 200 to 300 ms, followed by the object of the sentence (SONG, 40, two-character). Subjects were required to respond when the object of the sentence was presented on the screen (see Figure 1 for the experimental procedure).

The experiment was conducted in a quiet environment, and we conducted the experiment for each participant individually. During the experiment, subjects were seated in a chair with both eyes looking at the central point of the screen, 70 cm from the screen, with a horizontal and vertical viewing angle of <4°. All word pairs were presented in a randomized manner. Participants were asked to quickly and accurately determine whether the entire sentence could be understood. Participants were asked to press the “F” key on the keyboard with the left index finger if it was comprehensible and the “J” key with the right index finger if not. For the convenience of the participants, stickers with “✓” “✘” printed on them were placed on the “F” and “J” keys according to the size of the keyboard. The left and right hands, the stickers, and the keyboard were counterbalanced among the participants. To help them become familiar with the experimental process and requirements, participants completed practice trials before the formal experiment, using materials similar to those used in the experiment. The formal experimental phase consisted of 72 trials, with 7 short breaks between, and the whole experiment lasted for 12 min.

## 3 Data analysis and results

At the end of the experiment, we deleted all data from participants whose correct rates were less than 50%. Among them, two 5-year-old participants were deleted, leaving 10 remaining; a 6-year-old participant was deleted, leaving 11 remaining; a 7-year-old participant was deleted, leaving 11 remaining; all 8-year-old participants had an accuracy rate higher than 68%, so all data from twelve 8-year-old participants were retained. The results of the filler sentence test of all participants were excluded from the data analysis and sentences with incorrect responses were not included in the response time analysis. Furthermore, data with a response time outside of ±2.5 standard deviations for each condition were removed based on the age group of the participant. RTs were analyzed using linear mixed-effects modeling, with participant and item entered as random effects (all other variables were entered as fixed effects). The models of the accuracy rate data were analyzed using logistic regression. All analyses were conducted using R statistical software (R Core Team, 2023).

The model fitting procedure for each analysis started with a maximal model that included potential predictor variables as main effects. These included: age (5, 6, 7, and 8 years), sentence type(metaphorical, and literal), and salience(high, and low). For analyses using mixed effects modeling, item and person were entered as random effects. In addition, all models included all possible interactions between group and the other main effects. Categorical variables were dummy coded and all numerical predictor variables were standardized (using natural logs) and centered prior to analyses.

After constructing each maximal model, a backwards stepwise regression analysis was performed to identify the most plausible models for each measure using Akaike information criterion (AIC) values. No distinctions were made between main effects and interactions in this procedure. The predictor variable that had the least impact on the AIC values at each step was eliminated until only variables that significantly improved the fit were included. The analysis aimed to investigate the differences in processing metaphorical sentences and literal sentences with different salience by Chinese children of different ages. When there were interactions, emmeans package in R were used to show the specific performances on processing different sentences among children with different ages.

Descriptive statistics are presented in Table 2. For RTs, analyses showed that the main effect of age was not significant between the groups, Estimates = −0.129, *β* = −0.133, *t* = −1.13, *p* = 0.265. The main effect of sentence type was significant, Estimates = −0.184, β = −0.210, *t* = −2.81, *p* = 0.005, Cohen’s *d* = −2.808. The main effect of salience was marginally significant, Estimates = −0.131, *β* = −0.150, *t* = −1.94, *p* = 0.053, Cohen’s *d* = −1.942. The three-way interaction effect was significant, Estimates = −0.186, *β* = −0.166, *t* = −0.166, *p* = 0.010, Cohen’s *d* = −2.575. *Post hoc* comparisons between age groups for the interaction effect were performed. For 5-year-old children, there is a significant difference between metaphorical and literal sentences at low-saliency, Estimate = 0.184, *t* = 2.807, *p* = 0.027. For 6-year-old children, there is a significant difference between metaphorical and literal sentences, Estimate = 0.101, *t* = 2.204, *p* = 0.028. For 7-year-old children, there is a significant difference between metaphorical and literal sentences, Estimate = 0.102, *t* = 2.219, *p* = 0.028. For 8-year-old children, there is a marginally significant difference between metaphorical and literal sentences, Estimate = 0.085, *t* = 1.947, *p* = 0.053. For ACCs, analyses showed that the main effect of age was not significant between the groups, Estimate = 0.010, *p* = 0.179. The main effect of sentence type was not significant, Estimate = 0.050, *p* = 0.625. The main effect of salience was not significant, Estimate = 0.030, *p* = 0.641. The interaction effects were not significant, Estimate(s) ≤ 0.015, *p* ≥ 0.110.

**Table 2:** Average response time and accuracy rates.

Sentence type	Salience	**Age**	RT (ms)/Sdtime	**ACC**(%)/SdACC
Metaphorical sentence	Low-saliency	5-year-old	2302.68 (1293.15)	67.50 (47.03)
		6-year-old	2038.25 (853.04)	59.85 (49.21)
		7-year-old	2004.36 (697.84)	59.09 (49.35)
		8-year-old	1785.94 (677.86)	72.22 (44.95)
	High-saliency	5-year-old	2023.75 (828.36)	64.17 (48.15)
		6-year-old	2309.23 (1178.56)	58.33 (49.49)
		7-year-old	1894.05 (591.25)	60.61 (49.05)
		8-year-old	1827.64 (672.69)	62.50 (48.58)
Literal sentence	Low-saliency	5-year-old	1785.44 (818.80)	77.50 (41.93)
		6-year-old	1948.85 (895.99)	77.27 (42.07)
		7-year-old	1777.32 (482.68)	84.09 (36.72)
		8-year-old	1571.99 (544.82)	82.64 (38.01)
	High-saliency	5-year-old	2019.62 (1078.45)	68.33 (46.71)
		6-year-old	1990.75 (841.34)	73.48 (44.31)
		7-year-old	1857.22 (665.26)	76.52 (42.55)
		8-year-old	1708.14 (640.15)	72.92 (44.59)

**Figure 2:**
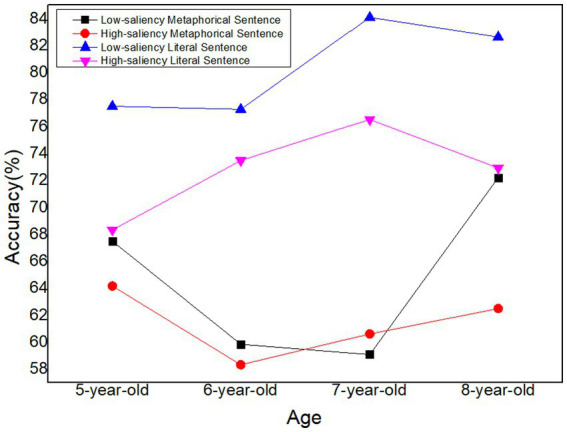
Accuracy rates for processing four types of experimental sentences in children of different ages.

**Figure 3:**
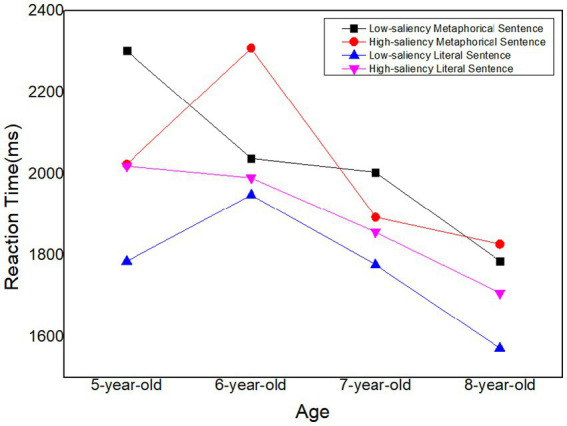
Response time to processing four types of experimental sentences in children of different ages.

Planned comparisons between age groups were performed. For 5-year-old children, there is no difference between metaphorical and literal sentences, Estimate = –0.0708, *t* = −1.294, *p* = 0.203. For 6-year-old children, there is a significant difference between metaphorical and literal sentences, Estimate = −0.163, *t* = 2.589, *p* = 0.013. For 7-year-old children, there is a significant difference between metaphorical and literal sentences, Estimate = −0.205, *t* = −3.198, *p* = 0.003. For 8-year-old children, there is a no difference between metaphorical and literal sentences, Estimate = −0.1042, *t* = −1.589, *p* = 0.119. The accuracy rate of the children’s processing of four types of experimental sentences at different ages and the trends in response time can be seen in Figures 2, 3.

## 4 Discussion

Utilizing a meaning decision task, the current study centered on drawing a comprehensive picture of the language development and cognitive processing of children’s metaphorical and literal sentences and elaborating on the similarities and differences in the processing pathways of metaphor among children at different ages. In addition, we found no significance on gender, so the present study controlled for gender as a variable.

### 4.1 The overall tendency of Chinese children’s metaphor comprehension: perception period, development period and rational decision period

The results of the current study demonstrated that children’s ability to understand metaphorical sentences developed gradually considering the increased ACC and decreased RT of almost every type of sentence among children at different ages (Figures 2, 3). That is, as children get older, their cognitive abilities and language proficiency continue to advance, which has become the important foundation for their comprehension of metaphorical sentences (Zou, 2012; Yuan, 2020). Based on our findings, 5 years old could be seen as a period of “metaphor perception” during which children have the intuition of difference between literal sentences and metaphor; 6 and 7-year-old children experience a period of “metaphor development” as a significant difference of accuracy in metaphor could be observed (*p* < 0.05); 8-year-old children are in a period of “rational decision” period during which they have relative low reaction and high accuracy.

First, we found that the effect of salience seems not so obvious than predicated overall for Chinese children aged 5–8, except for low-saliency sentences of 5-year-old children. 5-year-old in our study displayed a significant difference of reaction time in low-saliency sentences, and their processing time for metaphorical sentence (2302.68 ms) showed longer than literal sentence (1785.44 ms). Meanwhile at the same condition, there was no significant difference for accuracy. Children of 5-year-old could understand low-saliency metaphorical sentences as well as low-saliency literal ones while the latter with longer reaction time. Zhou et al. (2021) also found in their metaphor comprehension task, preschoolers, 4–5 year-old, understood literal and non-literal language equally well and they attributed the result to children’s relational reasoning mindset. Children at this stage are primed with language processing and world knowledge and are experiencing a gathering of each and every piece of knowledge to perceive the difference between low-saliency literal and metaphorical sentences. Because the connotations of the source domain bring children at that stage closer to their interactions with the world and closer to their bodily experiences, children at the age of 5 are more likely to learn earlier. Children can use this as a basis for forming initial hypotheses about the meaning of the target concept consisting of this source domain, which can help them understand the metaphors mapped to those target domains. Nevertheless, it is obvious to see that 5-year-old children employed longer time to process low-saliency metaphorical sentences than literal ones, which closely connected to semantic network. Semantic network is a type of data representation incorporating linguistic information that describes concepts or objects and the relationship or dependency between them (Nettleton, 2014). Literal sentences take less time to process due to their close semantic distance in the semantic network. So they could be easily understood by 5-year-old children whose vocabulary are limited. By contrast, metaphorical sentences’ source and target domains are in two different categories, and the semantic network is farther apart, thus requiring longer processing time and cognitive effort. In short, 5-year-old children have perceived the difference between low-saliency metaphorical sentence and literal ones, and they attempt to process metaphorical sentences from a perspective different from that of literal statements. That is, children at this stage have a certain level of embodied experience and vocabulary to perceive the differences between metaphorical and literal sentences. However, due to limited cognitive processing abilities, they require more time to identify metaphorical sentences. Therefore, we believe that metaphorical understanding in children at this stage falls within the metaphor perception period.

The second result is that we have noticed marked difference in 6 and 7 years old children upon their metaphorical comprehension and literal meaning processing according to accuracy. Children of 6 and 7 years old experienced better literal sentence processing than metaphorical ones. The reaction time for both low- and high saliency metaphorical sentences were longer than literal ones. At the age of 6–7, children have developed their theory of mind, which assists them to understand others’ different thoughts and feelings (Miller and Rose, 2009; Osterhaus and Koerber, 2021). However, their understanding is still more concrete and less abstract compared to older age groups. On one hand, literal sentences directly convey the speaker’s intended meaning without relying on abstract or metaphorical language. Children at this age are more adept at concrete thinking, which means they understand things more directly and literally. Literal sentences align well with their developing cognitive abilities and straightforward interpretation of the world. On the other hand, metaphorical language often involves understanding and interpreting abstract concepts or comparisons, which can be more challenging for children, but they still develop their cognitive flexibility. 6 and 7 years old children can understand simple metaphors, construct parsing categories based on perceptual foundations (Siltanen, 1986, 1990), and seem to begin processing metaphors word by word. And functional magnetic resonance imaging studies have also shown that the analogical reasoning and metaphor processing abilities of children at this age overlap in brain activation, show common underlying neural processes (Prat et al., 2012), and are similar to the brain activation areas of adults. The increased metaphorical ability can be explained, at least in part, by the increase in neural efficiency, that is, the increased functional connectivity within and between brain regions (Yin et al., 2016). Given that we infer that metaphorical comprehension of 6 and 7 years old children belong to development period.

Additionally, individuals in 8-year-old group exhibited the highest accuracy rate (72.22%) and the shortest response times (1785.94 ms) in low-saliency sentence comprehension. At this developmental stage, 8-year-old children demonstrate an enhanced ability to comprehend metaphors, attributing it to their capacity to activate source domains along with associated intentional schemas. This activation facilitates automatic and rapid mappings from the source domain to the target domain. Notably, the observed increase in processing speed suggests a corresponding increase in metaphorical understanding competence, as heightened processing speed contributes to the acquisition of new information.

Intriguingly, Figures 2, 3 revealed a negative growth in the accuracy rate and reaction time of high-saliency metaphorical sentences among 8-year-olds. We posit that this may indicate a reduced reliance on literal meaning in metaphorical comprehension, reflecting a deeper exploration of metaphorical meaning. Indeed, 8-year-old children engage their executive functions when processing complex linguistic phenomena, such as metaphor, as supported by studies (Best et al., 2009; Brydges et al., 2014). Their involvement in abstract reasoning, integrating information from both source and target domains, contributes to the understanding of metaphorical sentences. While the complexity of these cognitive processing procedures may lead to an increased likelihood of misjudgment and longer reaction times. In conclusion, we contend that 8-year-old children, during this deep-processing stage, attain a qualitatively new level of metaphor processing and belong to the rational decision period of metaphor mastery.

To fully explore the underlying processing mechanism of metaphor comprehension, we will illustrate our results from the perspectives of language development and cognitive processing in the following sections.

### 4.2 Language development and cognitive processing affect the metaphorical comprehension of Chinese children

To elaborate on the characteristics of Chinese children’s metaphors from the perspective of language development, we would analyze the potential reasons from two dimensions: vocabulary level and processing speed.

As an indispensable and essential component of language acquisition, vocabulary accounts for a vital component in verbal fluency and social interaction (Kelley and Poholik, 2023; Preethika and Gupta, 2023), serving as the primary source of constructing the external physical world and internal mental processes. From the perspective of vocabulary level, children’s performance on the metaphorical and literal sentences is progressive and proportional to age (Ferreira et al., 2023), presenting the way their flexible mapping between target domain and source domain and relatively high efficiency of identification of metaphorical and literal sentences. In a general view, children who were in higher vocabulary levels (e.g., 6- and 7-year-old children) tended to identify the words of the source domain and could map them to the relative target domain by combining the similarities of the two concepts as they demonstrated significant differences of accuracy and reaction time (*p* < 0.05). What should be noted is that 7-year-old children, who possess relatively high vocabulary levels, could identify the semantic meaning of literal sentences as they present higher accuracy compared with children at other different ages. For those who are aged 8, excessive vocabulary might mislead their judgments toward literal sentences that led to lower accuracy. The difference between the metaphor comprehension of 5- and 8-year-old children will be analyzed from the perspective of cognitive processing in 4.2.

Besides vocabulary level, processing speed is also a critical element in evaluating children’s comprehension of metaphorical and literal sentences. Based on the tendency in Figure 3, an interesting discovery was that children aged 6 took the longest time in processing high-saliency metaphorical sentences, which indicated that children at this age have difficulty in processing such sentences. A plausible reason might be that they are fully aware that high-saliency metaphorical sentences are not similar to other kinds of sentences. The time they cost might imply the internal mapping processing from the source domain to the target domain, which leads to low processing speed. Similarly, 5-year-old children began to realize the difference between low-saliency metaphorical and literal sentences (*p* < 0.05), which could be seen as proof of their awakenings to metaphors.

The underlying cognitive processing mechanism that might contribute to current results should be paid more attention, and we would explain it also from two perspectives: executive functions and abstract reasoning.

Consisting of response inhibition, working memory, and cognitive flexibility, executive functions (EF) develop rapidly during the preschool period and are considered as crucial contributors to general academic achievement (St Clair-Thompson and Gathercole, 2006; Diamond, 2013; Allan et al., 2014), including metaphor comprehension (Carriedo et al., 2016). The process of metaphor comprehension calls for the requirements of great abstraction and attention effort, which demands a high level of cognitive regulation—EF (Carriedo et al., 2016). To be specific, it involves (1) the activation or mapping of relative concepts from the source domain to the target domain and inhibition of irrelevant concepts (i.e., response inhibition); (2) bearing the information of the source domain which should be working with those in the target domain (i.e., working memory); and (3) changing the perspective flexibly between those two domains (i.e., cognitive flexibility). Based on our findings, 5-year-old children could recognize and distinguish metaphorical and literal sentences at low-saliency as there was a significant difference in reaction time between these two types of sentences (*p* < 0.05). We inferred that children at that age have begun to realize the concept of metaphor and might have combined or mixed the entities of source and target domains. As for 6-year-old children, which has been considered as a critical turn in the current study, they fully understood the differences between metaphorical and literal sentences and they tend to spend more energy or effort exploring the potential connection between source domain and target domain, which contributes to long RT and low processing speed in metaphor comprehension. Meanwhile, excessive cognitive cost might decrease their EF and result in low accuracy. An unexpected result of the accuracy of high-saliency metaphor sentences between 5- and 8-year-old children should not be ignored as it may reveal the unique cognitive processing mechanism of children in understanding metaphors. With higher EF, 8-year-old children may hold more irrelevant information in mind which may impact their response inhibition and further affect their working memory and cognitive flexibility, compared with 5-year-old children. However, we cannot simply conclude that 5-year-old children exhibit better comprehension of high-saliency metaphorical sentences than 8-year-old ones considering that there is no difference between metaphorical and literal sentences of ACC (*p* > 0.05). Therefore, there is a large chance that children who aged 5 would not distinguish these two types of sentences and process metaphorical sentences in the way they are employed in literal sentences. Nevertheless, this result should be treated with caution. Further research should employ a larger sample size to verify the findings of the current study.

In addition to EF, abstract reasoning has been widely known as the manipulation of self-generated thoughts, or thoughts that are not directly connected to the context, constrained by abstract elements that can be coordinated at one time (Hatcher et al., 1990; Marini and Case, 1994; Dumontheil, 2014). As grow older, children’s competencies to identify and distinguish metaphorical and literal sentences are strengthened. Till 8 years old, the cognitive efforts the children put in recognizing the sentence type we mentioned above were much lower, leading to the marginally significant difference between metaphorical and literal sentences in reaction time. Also, in accuracy, there was no significant difference was detected between these two types of sentences (*p* > 0.05), indicating that abstract reasoning no longer impacts children at this age, or it has been ignored unconsciously by 8-year-old children. For those aged 7, however, abstract reasoning may still play a vital role in decoding the semantic meaning of literal sentences as they used relatively low reaction time and obtained high accuracy.

## 5 Conclusion

Through a meaning decision task, this article investigates the difference, patterns, and salience of metaphorical and literal sentences processed by Chinese children of different ages. The study found that the metaphorical capacity of Chinese children increased with age, with a perception stage at age 5, a metaphorical development stage at age 6 and 7, and a rational decision stage of metaphorical ability at age 8. From then on, children can recall the knowledge of intention schema while activating the source domain and then automatically and rapidly map this knowledge to the target domain. At the same time, language development and cognitive processing influenced the metaphorical comprehension of Chinese children, typically, children of 8 years of age who had the highest correct rate and the shortest reaction time to process low-saliency metaphorical sentences. While 5-year-old children had the highest accuracy in high-salient metaphorical sentence and 6-year-old children got the longest reaction time to process sentence in high-saliency metaphor.

This study still has the following limitations. First of all, the sample size of this study is small, and more results of the study can be included in future studies. Secondly, due to the limited attention of children, in order to ensure the experimental validity, the study took 7 breaks during the experiment to ensure that children’s judgment of sentences was not affected by additional factors such as fatigue. Further studies should more directly explore the impact of vocabulary, executive functions, and so on given that they do not have external measures that could be used to sustain the claims about the putative mechanisms that explain the development of metaphor understanding for children. In addition, future studies can also use neuroscience or imaging methods to examine metaphor processing and its predictors in children of different ages to improve existing metaphor processing theories. The results of the study can also be compared with general data on brain development.

## Data availability statement

The datasets presented in this study can be found in online repositories. The names of the repository/repositories and accession number(s) can be found in the article/Supplementary material.

## Ethics statement

The studies involving human participants were reviewed and approved by the Ethics committee of School of Foreign Languages, China University of Petroleum (East China). Written informed consent to participate in this study was provided by the participants’ legal guardian/next of kin.

## Author contributions

LC: Conceptualization, Writing – original draft, Data curation, Formal analysis, Funding acquisition. TZ: Conceptualization, Data curation, Supervision, Writing – review & editing. LZ: Formal analysis, Writing – review & editing. YL: Conceptualization, Data curation, Writing – review & editing. SY: Formal analysis, Writing – review & editing. YP: Formal analysis, Validation, Writing – review & editing. YG: Supervision, Validation, Visualization, Writing – review & editing. PW: Validation, Writing – review & editing.

## Footnotes

http://www.360doc.com/content/18/0405/10/40814224_743008411.shtml^1^

^2^Since children have not yet understood the concepts of conventionality and typicality, only children’s familiarity with the sentences is tested.

^3^According to Piaget, the age of children’s preoperational stage is generally between 2 and 7 years. Given that children do not establish “physical-mental metaphors” until the age of 5 years (Waggoner and Palermo 1989; Pan and Zhou, 2021), and they can basically recognize Chinese characters by the age of 5, so the age range of the subjects was chosen to be 5–8 years.

^4^Although studies by Kogan and Chadrow (1986) and Willinger et al. (2017) found no effect of gender on children’s metaphor comprehension, the present study controlled for gender as a variable.

^5^Song typeface is a kind of Chinese font which is adapted to the printing technique. The stroke thickness changes, and is generally horizontal thin vertical thick, the end has a decorative part (that is, “foot” or “serif”), point, skim, tick, hook and other strokes have a tip, belonging to the serif font (serif), often used in books, magazines, newspapers printing body typesetting.
