# Supplementary material for: An empirical study on the development of metaphorical comprehension of Chinese children
Source: Front Psychol. 2024 Jan 8;14:1254129. doi: 10.3389/fpsyg.2023.1254129 (PMC10800952; doi:10.3389/fpsyg.2023.1254129)
Supplement: Supplementary file 1 [file Data_Sheet_1.pdf]

Supplementary File in Chinese

1. Experimental material

display1	display2	display3
思念	是	潮水
梦想	是	翅膀
草坪	是	地毯
大树	是	空调
海洋	是	麦田
云朵	是	棉花
太阳	是	皮球
小脸	是	苹果
生活	是	舞台
荷叶	是	小伞
星星	是	眼睛
雨水	是	眼泪
智慧	是	钥匙
爸爸	是	超人
眼睛	是	窗户
困难	是	敌人
彩虹	是	拱桥
儿童	是	花朵
祖国	是	花园
时间	是	金钱
书籍	是	朋友
孩子	是	小苗
学校	是	摇篮
迪迦	是	英雄
老师	是	园丁
警察	是	保护
孝顺	是	传统
发明	是	创造
劳动	是	光荣
运动	是	健康
成长	是	快乐
问候	是	礼貌
团队	是	力量
诚实	是	美德
分享	是	善良
学生	是	未来
家庭	是	温暖
蔬菜	是	营养
小狗	是	动物
电脑	是	工具
教师	是	职业

宝宝	是	孩子
唐僧	是	和尚
长江	是	河流
妈妈	是	女性
大脑	是	器官
白菜	是	蔬菜
西瓜	是	水果
积木	是	玩具
嫦娥	是	仙女
花园	是	班级
大象	是	鼻子
米饭	是	蛋糕
电视	是	动画
小熊	是	公园
玩具	是	花朵
日历	是	报纸
桌子	是	钢笔
书本	是	泪水
大楼	是	鱼肉
皮球	是	气球
汽车	是	面包
家具	是	书本
苹果	是	果汁
瀑布	是	水杯
手机	是	夹子
学校	是	同学
茶叶	是	篮球
鸭子	是	香蕉
小羊	是	小草
蝴蝶	是	小河
青蛙	是	小鸟
镜子	是	心情
饺子	是	音乐
牛奶	是	饮料
小猫	是	春天

2.Practice material

display1	display2	display3
昨天	是	眼镜
白雪	是	白糖
人生	是	旅行
成功	是	光荣
松鼠	是	动物
天气	是	笔记

Supplementary File in English

Experimental material

Display1	Display2	Display3
Longing	is	tide
Dream	is	wing
Lawn	is	carpet
Tree	is	air conditioner
Sea	is	cornfield
Cloud	is	cotton
Sun	is	ball
Face	is	apple
Life	is	stage
Lotus leaf	is	umbrella
Star	is	eye
Rainwater	is	tear
Wisdom	is	key
Father	is	superman
Eye	is	Window
Difficulty	is	enemy
Rainbow	is	arch
Child	is	flower
Motherland	is	garden
Time	is	money
Book	is	friend
Kid	is	seedling
School	is	cradle
Tiga	is	hero
Teacher	is	gardener
Police	is	protection
Filial piety	is	tradition
Invention	is	creation
Work	is	glory
Exercise	is	health
Growth	is	joy
Greeting	is	politeness
Unity	is	power
Honesty	is	virtue
Sharing	is	kindness
Student	is	future
Family	is	warmth
Vegetable	is	nutrition
Dog	is	animal

Computer	is	tool
Teacher	is	15
Baby	is	kid
Xuan Zang	is	monk
Yangtze River	is	river
Mother	is	woman
Brain	is	organ
Cabbage	is	vegetable
Watermelon	is	fruit
Building blocks	are	toys
Chang'e	is	fairy
Garden	is	class
Elephant	is	nose
Rice	is	cake
Television	is	cartoon
Bear	is	park
Toy	is	flower
Calendar	is	newspaper
Table	is	pen
Book	is	tear
Building	is	fish
Ball	is	balloon
Car	is	bread
Furniture	is	book
Apple	is	juice
Waterfall	is	cup
Mobile phone	is	clip
School	is	classmate
Tea	is	basketball
Duck	is	banana
Lamb	is	grass
Butterfly	is	rivulet
Frog	is	bird
Mirror	is	mood
Dumplings	are	music
Milk	is	beverage
Kitten	is	spring

2. Practice material

Display 1	Display 2	Display 3
Yesterday	is	glasses
Snow	is	sugar
Life	is	journey

Success	is	honor
Squirrel	is	animal
Weather	is	note
